# Alternative Splicing Regulation During Light-Induced Germination of *Arabidopsis thaliana* Seeds

**DOI:** 10.3389/fpls.2019.01076

**Published:** 2019-09-10

**Authors:** Rocío Soledad Tognacca, Lucas Servi, Carlos Esteban Hernando, Maite Saura-Sanchez, Marcelo Javier Yanovsky, Ezequiel Petrillo, Javier Francisco Botto

**Affiliations:** ^1^Consejo Nacional de Investigaciones Científicas y Técnicas, Instituto de Investigaciones Fisiológicas y Ecológicas Vinculadas a la Agricultura (IFEVA), Facultad de Agronomía, Universidad de Buenos Aires, Buenos Aires, Argentina; ^2^Facultad de Ciencias Exactas y Naturales, Departamento de Fisiología, Biología Molecular y Celular and CONICET-UBA, Instituto de Fisiología, Biología Molecular y Neurociencias (IFIBYNE), Universidad de Buenos Aires (UBA), Buenos Aires, Argentina; ^3^Fundación Instituto Leloir, IIBBA-CONICET, Buenos Aires, Argentina

**Keywords:** dormancy, germination, light, phytochrome B (phyB), alternative splicing (AS), Arabidopsis

## Abstract

Seed dormancy and germination are relevant processes for a successful seedling establishment in the field. Light is one of the most important environmental factors involved in the relief of dormancy to promote seed germination. In *Arabidopsis thaliana* seeds, phytochrome photoreceptors tightly regulate gene expression at different levels. The contribution of alternative splicing (AS) regulation in the photocontrol of seed germination is still unknown. The aim of this work is to study gene expression modulated by light during germination of *A. thaliana* seeds, with focus on AS changes. Hence, we evaluated transcriptome-wide changes in stratified seeds irradiated with a pulse of red (Rp) or far-red (FRp) by RNA sequencing (RNA-seq). Our results show that the Rp changes the expression of ∼20% of the transcriptome and modifies the AS pattern of 226 genes associated with mRNA processing, RNA splicing, and mRNA metabolic processes. We further confirmed these effects for some of the affected AS events. Interestingly, the reverse transcriptase–polymerase chain reaction (RT–PCR) analyses show that the Rp modulates the AS of splicing-related factors (*At-SR30*, *At-RS31a*, *At-RS31*, and *At-U2AF65A*), a light-signaling component (*At-PIF6*), and a dormancy-related gene (*At-DRM1*). Furthermore, while the phytochrome B (phyB) is responsible for the AS pattern changes of *At-U2AF65A* and *At-PIF6*, the regulation of the other AS events is independent of this photoreceptor. We conclude that (i) Rp triggers AS changes in some splicing factors, light-signaling components, and dormancy/germination regulators; (ii) phyB modulates only some of these AS events; and (iii) AS events are regulated by R and FR light, but this regulation is not directly associated with the intensity of germination response. These data will help in boosting research in the splicing field and our understanding about the role of this mechanism during the photocontrol of seed germination.

## Introduction

Seed dormancy is a developmental checkpoint that allows plants to regulate when and where they grow. Temperature, light, and nitrates are the most relevant environmental factors regulating the relief of seed dormancy to promote seed germination (Benech-Arnold et al., 2000). These cues can trigger molecular responses including hormone signaling, mainly those of abscisic acid (ABA) and gibberellin (GA). The balance between the contents and sensitivity of these hormones is key for the regulation of the dormancy status of the seeds. ABA promotes primary dormancy induction and later maintenance, whereas GA promotes seed germination. Environmental signals regulate this balance by modifying the expression of metabolic enzymes as well as those of positive and negative regulators of both hormones, many of which are feedback regulated (Finkelstein et al., 2008).

Light has been one of the most characterized factors regulating the relief of dormancy. Phytochromes are the best-known photoreceptors perceiving red (R) and far-red (FR) light. They are synthesized in its inactive form, Pr (with maximum absorption in R), and are photo-converted into their active form, Pfr (with maximum absorption in FR). The *Arabidopsis* genome encodes five phytochromes, named phyA to phyE. Among them, phytochrome B (phyB) has a prominent role as the main photoreceptor regulating the R/FR reversible response (Shinomura et al., 1994; Botto et al., 1995), and phyD and phyE can contribute to this regulation in *phyA/phyB* double mutant seeds (Hennig et al., 2002; Arana et al., 2014), suggesting some redundancy in phytochrome functions in the R-light-mediated seed germination. In the soils, weed seeds change their light sensitivity according with after-ripening and burial conditions (Scopel et al., 1991; Botto et al., 1998a; Botto et al., 1998b; Botto et al., 2000), being phyA responsible for the detection of very brief light stimulus-promoting seed germination (Botto et al., 1996; Shinomura et al., 1996).

Light tightly regulates the expression (transcript levels) of thousands of genes (Casal and Yanovsky, 2005; Galvao and Fankhauser, 2015) and also many other layers of gene expression such as mRNA splicing, translation, and stability (Mano et al., 1999; Yakir et al., 2007; Simpson et al., 2008; Jung et al., 2009; Juntawong and Bailey-Serres, 2012; Liu et al., 2012; Paik et al., 2012; Liu et al., 2013; Petrillo et al., 2014; Shikata et al., 2014; Tsai et al., 2014; Wang et al., 2014; Wu et al., 2014; Mancini et al., 2016). RNA splicing is a co-transcriptional molecular event that is carried out by a macromolecular complex called spliceosome. Alternative splicing (AS) is the process that generates multiple transcripts from a single gene by using different combinations of available splice sites. AS leads to different outcomes and produces transcripts encoding for proteins that may have altered or lost function. Several investigations have demonstrated the importance of AS in processes like photosynthesis, defense responses, circadian clock, hormone signaling, flowering time, and metabolism (Lightfoot et al., 2008; Matsumura et al., 2009; Sanchez et al., 2010; Martin-Trillo et al., 2011; James et al., 2012; Jones et al., 2012; Rosloski et al., 2013).

AS is also relevant during the early (Fouquet et al., 2011) and late stages of embryo development (Sugliani et al., 2010). At the seed level, the work by Srinivasan et al. (2016) analyzed the AS during *Arabidopsis* seed development at a global level, both before and after seed desiccation. They identified 4,003 genes that are alternatively spliced, and 1,408 of those genes showing a differential pattern of splicing between both stages. Remarkably, most of these alternatively spliced transcripts had not been found in other tissues. More recently, another report highlights the relevance of AS in *Arabidopsis* seeds (Narsai et al., 2017). These authors analyzed the dynamics of gene expression over 10 developmental time points during seed germination and identified 620 genes undergoing AS. The regulation of these AS events during seed germination is time specific and/or tissue specific (Narsai et al., 2017). Interestingly, they also found complex variations in the relative abundance of *PIF6* (*PHYTOCHROME INTERACTING FACTOR 6*, *AT3G62090*) isoforms, as previously demonstrated (Penfield et al., 2010). PIF6 transcription factor is expressed during seed development, and its expression is dramatically reduced during imbibition. It has four known AS isoforms (Narsai et al., 2017), one of them originating from an exon skipping (ES) event that creates a premature stop codon and encodes for a protein that lacks the DNA-binding domain (Penfield et al., 2010). As expected, the overexpression of this *PIF6* isoform reduces seed dormancy (Penfield et al., 2010). *DOG1* (*DELAY OF GERMINATION1*, AT5G45830), another main regulator of seed germination, is also affected at the AS level. DOG1 protein accumulates during seed maturation, and its abundance in freshly harvested seeds determines the dormancy status of the seed (Graeber et al., 2014). *DOG1* transcripts are extensively alternatively spliced, giving place to five AS isoforms that are all functional but unstable if not expressed in combination (Bentsink et al., 2006; Nakabayashi et al., 2012; Nakabayashi et al., 2015). These findings clearly show the importance of AS control in the regulation of seed germination.

Photosensory-protein pathways are not the only way to sense light and regulate gene expression accordingly. Petrillo et al. (2014) have shown that *At-*RS31 (*ARGININE/SERINE-RICH SPLICING FACTOR 31*, AT3G61860), *At-SR30* (*ARGININE/SERINE-RICH SPLICING FACTOR 30*, *AT1G09140*), and *At-U2AF65A* (*U2 SNRNP AUXILIARY FACTOR*, *AT4G36690*) AS patterns are modulated by different light conditions through retrograde signals arising from chloroplasts. They demonstrated that the chloroplast, which is able to sense and to communicate light signals to the nucleus, is the main actor triggering the AS changes in response to light (Petrillo et al., 2014). All these experimental evidences suggest that AS is a relevant mechanism even though there is still no information available about how light-promoting seed germination affects this process in *Arabidopsis thaliana* seeds. Here, we showed that (i) red pulse (Rp) triggers AS changes in some splicing factors, light-signaling components, and dormancy/germination regulators; and (ii) phyB is involved in the regulation of only some of these AS events. Furthermore, (iii) AS events are regulated by R and FR light, but this regulation is not directly associated with the intensity of germination response. We conclude that AS is a source of gene expression diversity, and a proper regulation of this process might be of key relevance for seed germination modulation under different light conditions.

## Materials and Methods

### Plant Material and Growth Conditions

*A. thaliana* plants were grown under long day conditions [16-h L/8-h D, photosynthetically active radiation (PAR) = 100 *µmol·m* ^−2^ *·s* ^−1^] with an average temperature of 21 ± 2°C. Plants were grown together, and their mature seeds were harvested at the same time to avoid differences in post-maturation, which can affect seed germination. Seeds of each genotype were harvested as a single bulk that consisted of at least five plants. Seeds were stored in tubes with small holes inside a closed box and maintained in darkness with silica gel at 4°C until the experiments were performed. *A. thaliana* Columbia-0 (Col-0) and Landsberg *erecta* (L*er*) were used as wild type (WT). Seeds of *phyB-9* (Col-0 background) and *phyB-5* (L*er* background) (218790) were obtained from the ABRC (www.arabidopsis.org/abrc/).

### Germination Conditions and Light Treatments

Samples of 20 seeds per genotype were sown in clear plastic boxes, each containing 10 ml of 0.8% (w/v) agar in demineralized water. To establish a minimum and equal photo-equilibrium, seeds were imbibed for 2 h in darkness and then irradiated for 20 min with a saturated far-red pulse (FRp; calculated Pfr/P = 0.03, 42 *µmol·m* ^−2^ *·s* ^−1^) in order to minimize the quantities of Pfr that formed during their development in the mother plant. Seeds were then stratified at 5°C in darkness for 3 days, prior to the 20 min with a saturated Rp (calculated Pfr/P = 0.87, 0.05 *µmol·m* ^−2^ *·s* ^−1^) or FRp. After light treatments, the boxes containing the seeds were wrapped again with black plastic bags and incubated at 25°C for 3 days before germination was determined. The criterion for germination was the emergence of the radicle.

For experiments with hormones, seeds were sown in clear plastic boxes, each containing filter papers imbibed with 750 µl of fluridone 100 µM (Sigma-Aldrich, Steinheim, Germany) or ABA 1 µM supplemented with fluridone 100 µM (Ibarra et al., 2016) until the end of the experiment. We have previously performed calibration curves to determine the optimal ABA concentration to counteract the promotion of germination triggered by fluridone (**Supplementary Figure 1**).

### cDNA Library Preparation and High-Throughput Sequencing

Seed samples were sown in clear plastic boxes, each containing 10 ml of 0.8% (w/v) agar in de-mineralized water. Three biological replicates of each condition were collected 12 h after the corresponding R and FR light pulses. After sampling, seeds were immediately frozen in liquid nitrogen and stored at −80°C. RNA was extracted using the Spectrum Plant Total RNA Kit (Sigma-Aldrich, Steinheim, Germany) according to manufacturer’s protocol. To estimate the concentration and quality of the samples, spectrophotometry and agarose gel were used, respectively. RNA samples were processed at the Instituto de Agrobiotecnología de Rosario (INDEAR, Rosario, Argentina). Samples were pooled to create six multiplexed DNA libraries, which were pair-end sequenced with an Illumina HiSeq 1500.

### Processing of RNA Sequencing Reads

Sequence reads were aligned with the *A. thaliana* genome TAIR10 (Langmead et al., 2009) with TopHat v2.1.1 (Trapnell et al., 2009) with default parameters, except in the case of the maximum intron length parameter, which was set at 5,000. Count tables for the different feature levels were obtained from bam files using custom R scripts and considering the TAIR10 transcriptome.

### Differential Gene Expression Analysis

Differential gene expression analysis was conducted for genes whose expression was above a minimum threshold level [>10 reads and a read density (RD) >0.05] in at least one experimental condition. RD was computed as the number of reads in each gene divided by its effective width. The term effective width corresponds to the sum of the length of all the exons of a given gene. Differential gene expression was estimated using the edgeR package version 3.4.2 (Robinson et al., 2010), and resulting *p*-values were adjusted using a false discovery rate (FDR) criterion (Benjamini and Hochberg, 1995). Genes with FDR values lower than 0.05 and an absolute fold change >1.5 were considered to be differentially expressed (DE). This dataset was labeled as DE genes (**Supplementary Table 1**).

### Differential AS Analysis

For the analysis of differential AS, multiexonic genes were partitioned into features defined as “bins,” corresponding to exonic, intronic, and alternatively spliced regions. We labeled these three kinds of bins as exon bins, intron bins, or AS bins, respectively. In addition, AS bins were further classified as ES, alternative 5′ splice site (Alt5′SS), alternative 3′ splice site (Alt3′SS), and intron retention (IR). Bins with three or more different AS events in the same subgenic region were labeled as multiple. Read summarization was performed at those three levels: exon, intron, and AS bins. These datasets were then filtered according to several criteria applied at the gene and bin levels. First, defined subgenic regions (i.e., bins) were considered for differential AS analysis only if the genes with which they are associated with were expressed above a minimum threshold level (more than 10 reads per gene and RD > 0.05) in all experimental conditions. Next, bins were considered for differential AS analysis only if they had more than five reads and an RD bin/RD gene ratio >0.05, in at least one experimental condition. After these filters were applied, reads summarized at the bin level were normalized to the read counts of their corresponding gene. This was done to avoid the influence of changes in gene expression on the differential AS analysis at the bin level. Then, similarly to the approach used for the differential expression analysis, differential AS analysis was conducted at the bin levels using the edgeR package version 3.4.2. Bins with FDR values lower than 0.15 were considered to undergo differential AS. Finally, we restricted the selection of AS bins to those bins for which differential AS analysis was supported by expected changes in the numbers of splice junctions. In order to do this, we obtained information on the number of reads associated with each splice junction, both annotated and novel. Junction coordinates were extracted from gap containing aligned reads. Junctions with fewer than five reads were discarded. We then computed the metrics PSI (percent spliced-in) and PIR (percent IR), which were used as a final filtering criteria for the AS analysis. PSI was defined as the percentage of the number of junction reads supporting bin inclusion relative to the combined number of reads supporting inclusion and exclusion (Pervouchine et al., 2013). PSI values were computed for ES, Alt5′SS, and Alt3′SS. PIR values, calculated as previously described (Braunschweig et al., 2014), were used for the IR analysis. Briefly, PIR is defined for each experimental condition as the percentage of the number of reads supporting IR (E1I + IE2) relative to the combined number of reads supporting IR and exclusion (E1I + IE2 + 2 exclusion junction [JE1E2]), where E is the exonic bin, I the intronic bin, and J the junction (Mancini et al., 2016). AS, exon, and intron bins were considered to be differentially spliced (DS) if, in addition to fulfilling the filtering criteria described above, the difference in PSI or PIR between experimental conditions was >0.5%. Bins corresponding to alternatively spliced regions identified through novel splice junctions were considered to be differentially alternatively spliced if there was a difference in the PSI value larger than 10% between experimental conditions. This dataset was labeled as DS genes (**Supplementary Table 1**).

### Gene Ontology Analysis

Gene ontology (GO) terms assignment for the DE genes and DS genes datasets were obtained using the BioMaps tool from the virtual plant software (http://virtualplant.bio.nyu.edu/cgi-bin/vpweb/). An enrichment test was performed for the following categories: BP (biological process), MF (molecular function), and CC (cellular component). *p*-values were obtained using the Fisher exact test and corrected for multiple testing using FDR. The enrichment factor (EF) was estimated as the ratio between the proportion of genes associated with a particular GO category present in the dataset under analysis, relative to the number of genes in this category in the whole genome (**Supplementary Table 1**). Bubble plots were generated, using a custom script written in R language, for all those categories for which the adjusted *p*-value was lower than 0.01 in at least one dataset.

### Semi-Quantitative Reverse Transcriptase–Polymerase Chain Reaction

Seed samples were sown in clear plastic boxes, each containing 10 ml of 0.8% (w/v) agar in de-mineralized water. Three biological replicates of each condition were collected 12 h after the corresponding R and FR light pulses. After sampling, seeds were immediately frozen in liquid nitrogen and stored at −80°C. RNA was extracted using the Spectrum Plant Total RNA Kit (Sigma-Aldrich, Steinheim, Germany) according to manufacturer’s protocol. cDNA derived from the extracted RNA was synthesized using M-MLV reverse transcriptase (Promega, Madison, WI, USA) and oligo-dT primers. polymerase chain reaction (PCR) analyses were conducted using a mix with Taq DNA Pol (Inbio Highway, Tandil, Buenos Aires, Argentina), 10× buffer, 10× polyvinylpyrrolidone (PVP), 25 mM of MgCl_2_, 10 mM of dNTPs, and gene-specific primers according to the manufacturer’s instructions. The PCR program was as follows: 95°C for 3 min and the respective number of cycles (28–31) at 95°C for 30 s, 60°C for 30 s, and 72°C for 1.5 min. The amplified products were resolved by 1% or 2% agarose gel electrophoreses. The SI, defined as the abundance of the longest splicing isoform relative to the levels of all possible isoforms, was calculated from the relative levels of the corresponding reverse transcriptase (RT)–PCR products quantified using densitometry by the ImageJ software (https://imagej.net/Welcome). Gene models and gel images are shown in **Supplementary File 1**. The specific primers used are described in **Supplementary Table 2**.

### Statistical Analysis

To test for significant differences in the response of the seeds, we conducted two-way analyses of variance (ANOVAs) for each WT and mutant group, using the angular transformation of the percentage of germination and the InfoStat Software version 2017 (Grupo InfoStat, FCA, Universidad Nacional de Córdoba, Argentina). Fisher post-test was used to test differences between genotypes, when significant treatment-by-genotype interactions were observed.

To test for significant differences in the splicing index (SI), we also conducted two-way ANOVAs using the same software, and Fisher post-test was used to test for differences, when significant treatment-by-genotype interactions were observed. When indicated, we used Student’s *t*-test.

## Results

### Seed Germination Induced by Red Light Modulates the AS Pattern of 226 genes

**Figure 1:**
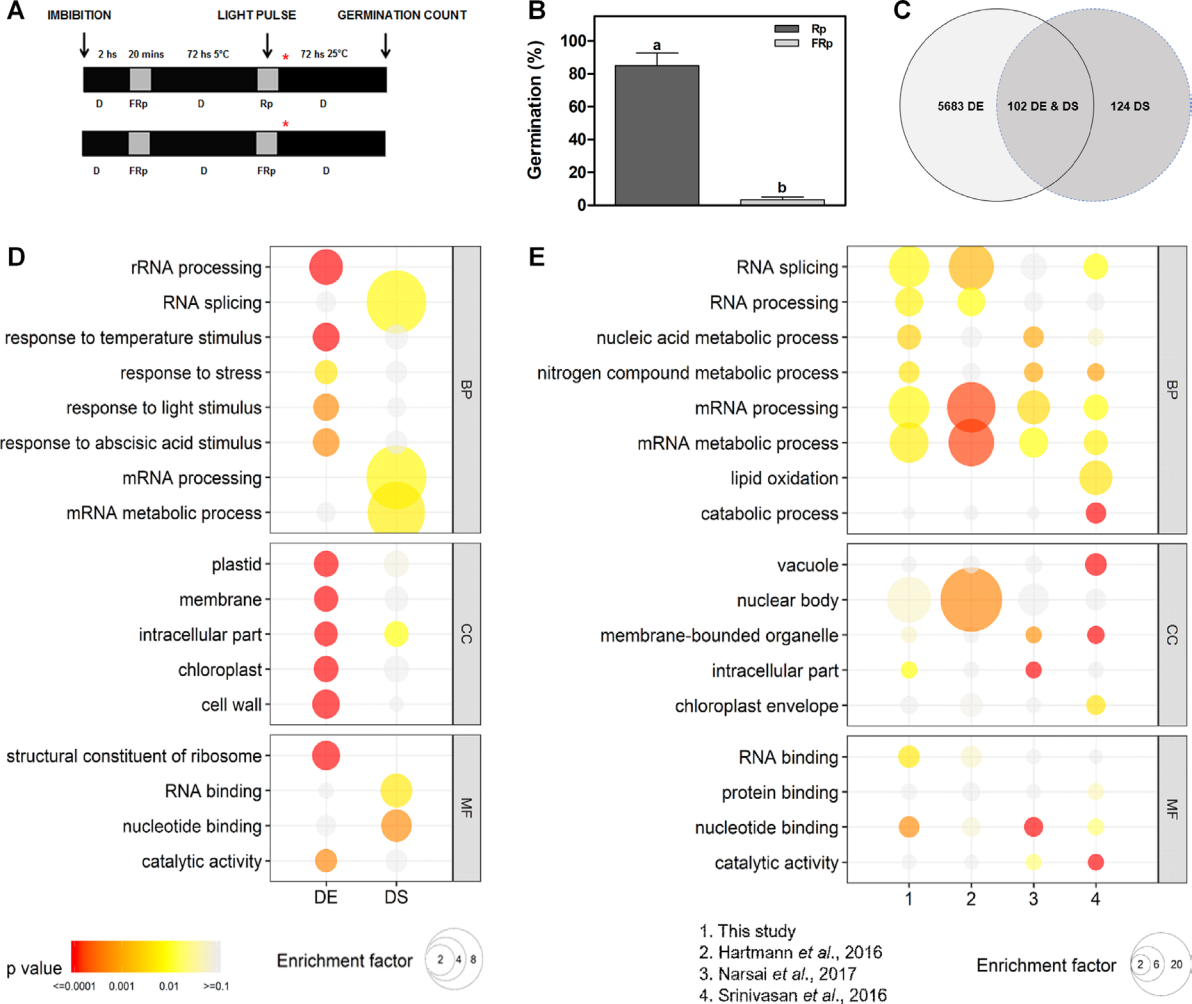
Germination induced by a red pulse modifies the alternative splicing pattern of 226 genes in Col-0 seeds. **(A)** Scheme of the experimental protocol. Col-0 seeds were irradiated with an Rp or an FRp after 3 days of chilling, and samples for RNA isolation were collected 12 h after the light pulse. Asterisk shows sampling point for RNA extractions. **(B)** Germination percentage of Col-0 seeds irradiated with an Rp (85%) or an FRp. Each bar represents mean ± SE (*n* = 3). Significant differences between means are shown by different letters (*p* < 0.05 by Student’s *t*-test). **(C)** Overlap between genes differentially affected by light at the expression level (DE genes) and genes regulated by light at the AS level (DS genes). **(D)** GO enrichment analysis comparing the DE and DS genes of our study. **(E)** GO enrichment analysis comparing DS genes between our study, and the works by Hartmann et al. (2016), Narsai et al. (2017), and Srinivasan et al. (2016). For panels D and E, GO was evaluated at three different levels: biological processes (BP), cellular component (CC), and molecular function (MF). The color gradient represents adjusted *p*-values, and the differences in bubble size correlate with the enrichment factor. Only those categories showing a statistically significant enrichment at either gene expression or AS level are depicted. AS, alternative splicing; Col-0, Columbia-0; DE, differentially expressed; DS, differentially spliced; FRp, far-red pulse; GO, gene ontology; Rp, red pulse.

WT Col-0 seeds were imbibed in darkness at 5°C for 3 days prior to irradiation with an Rp or an FRp. Germination was counted after 3 days at 25°C in darkness (**Figure 1A**). The Rp significantly induces seed germination ∼ 85% in Col-0 seeds, while the FRp does not promote seed germination (**Figure 1B**), indicating that the phytochrome system is involved in this response. We used this seed population to evaluate changes at the transcriptome level triggered by light, with special focus in AS modulation. Seed samples for RNA isolation were collected 12 h after the light treatments (**Figure 1A**), and R light effects on mRNA levels and AS were analyzed (**Supplementary Table 1**). We identified a total of 5,785 genes whose mRNA levels are significantly affected, either increased or decreased, more than 1.5-fold (FDR < 0.05) in response to the Rp (**Figure 1C** and **Supplementary Table 1**). We defined this group as DE genes. Interestingly, DE genes are enriched in genes associated with response to temperature, light, and ABA stimuli (**Figure 1D** and **Supplementary Table 1**). We then evaluated the effects of R light on AS and identified a total of 226 genes with AS events that are regulated by the light pulse (**Figure 1C** and **Supplementary Table 1**). We defined this group as DS genes. Less than half of the genes whose AS patterns are affected by R light show alterations at the total mRNA levels too (102 genes, **Figure 1C**). We also observed a strong enrichment in GO categories associated with mRNA processing, RNA splicing, and mRNA metabolic processes among these DS genes (**Figure 1D** and **Supplementary Table 1**). These categories are not enriched among the genes whose mRNA levels, rather than AS patterns, are affected by R light, supporting the idea that light regulates AS patterns mostly through its effect on the AS of splicing factors themselves (**Figure 1C** and **Supplementary Table 1**).

Additionally, we performed a comparative transcriptomic analysis to evaluate if differentially alternatively spliced genes identified here, as responsive to an Rp that induced germination, were also regulated in other developmental processes or conditions, like seed maturation (Srinivasan et al., 2016), germination induced by a white light/dark photoperiod (Narsai et al., 2017), and/or seedling de-etiolation (Hartmann et al., 2016). We found two common genes affected at the AS level in the four transcriptome studies (*At-RS41* and *At-HYP1*), but a higher number of common genes are found when comparing different combinations of specific transcriptomes with those in our study (**Supplementary Table 1**). We also observed a strong enrichment in GO categories associated with RNA splicing, RNA processing, mRNA processing, and mRNA metabolic processes among these different physiological processes (**Figure 1E** and **Supplementary Table 1**). These data are in agreement with previous studies showing that genes related to RNA metabolism are among the most affected by AS (Syed et al., 2012; Reddy et al., 2013).

### Red Pulse Reduces the SI of *At-SR30*, *At-RS31a*, *At-RS31*, and *At-U2AF65A*

**Figure 2:**
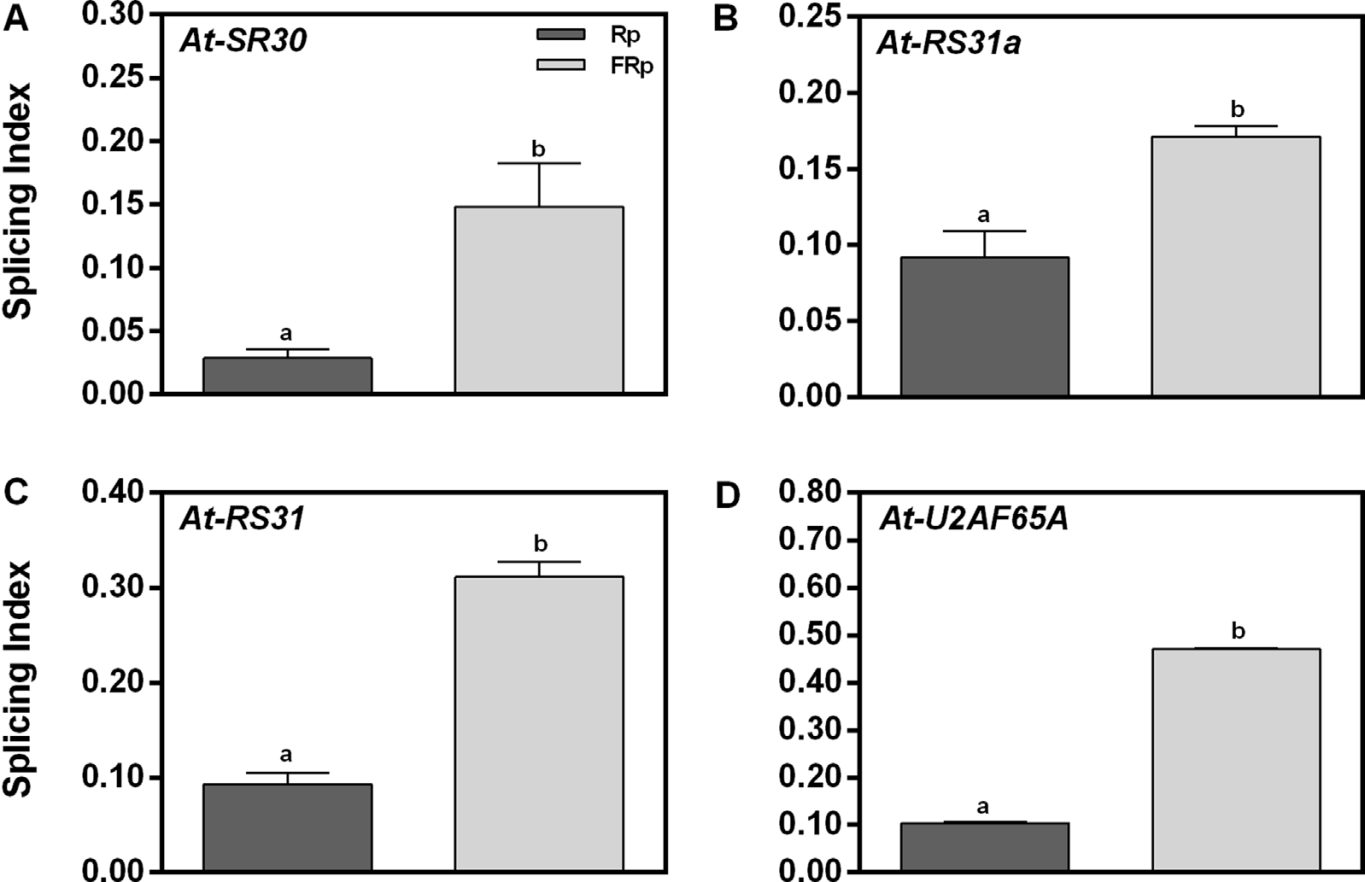
Germination and alternative splicing regulation by red/far-red light. **(A-D)** Germination induced by an Rp reduces the splicing index (SI) of *At-U2AF65A*, *At-SR30*, *At-RS31*, and *At-RS31a* in Col-0 seeds. We selected these genes from the RNA-seq data based on two simultaneous criteria: (1) genes involved in RNA splicing and (2) genes previously documented to have AS (Petrillo et al., 2014; Shikata et al., 2014). RNA Col-0 samples were harvested 12 h after the Rp or FRp. AS alterations as SI in response to the Rp or FRp are shown. Gene expression levels were analyzed by RT–PCR, and each PCR product was quantified by densitometry using ImageJ. Each bar represents mean ± SE (*n* = 3). Significant differences between means are shown by different letters (*p* < 0.05 by Student’s *t*-test). AS, alternative splicing; Col-0, Columbia-0; FRp, far-red pulse; Rp, red pulse; RT–PCR, reverse transcriptase–polymerase chain reaction.

To validate the RNA sequencing (RNA-seq) data, we selected four genes of our interest and studied their responses by RT–PCR: (a) *At-SR30* and *At-RS31*, which are among the down-regulated and AS affected genes (DE and DS) according to the RNA-seq (**Supplementary Table 1**), and (b) *At-U2AF65A* and *At-RS31a*, which belong to the down-regulated category (DE) but are known to be light-responsive events in seedlings (Petrillo et al., 2014). All these candidate genes are related to the splicing process itself: three are SR genes (*At-SR30*, *At-RS31a* and *At-RS31*) and one is an auxiliary splicing factor (*At-U2AF65A*). We used the SI, defined as the abundance of the longest splicing isoform relative to the levels of all possible isoforms, as the parameter to evaluate AS changes induced by the Rp. **Figure 2** shows that the Rp reduces the SI of *At-SR30*, *At-RS31a*, *At-RS31*, and *At-U2AF65A* between 2- and 5-folds than does the FRp. These results confirm that the AS patterns of these events are modulated by red light when seed germination is induced. Moreover, these results suggest that the total number of affected AS events by red light in germinating seeds is probably higher than those considered in our list (**Supplementary Table 1**). Further research in this direction is needed to know the actual impact of AS in gene expression regulation by red light during seed germination.

### Phytochrome B Regulates the AS Pattern of *At-PIF6* and *At-U2AF65A*

**Figure 3:**
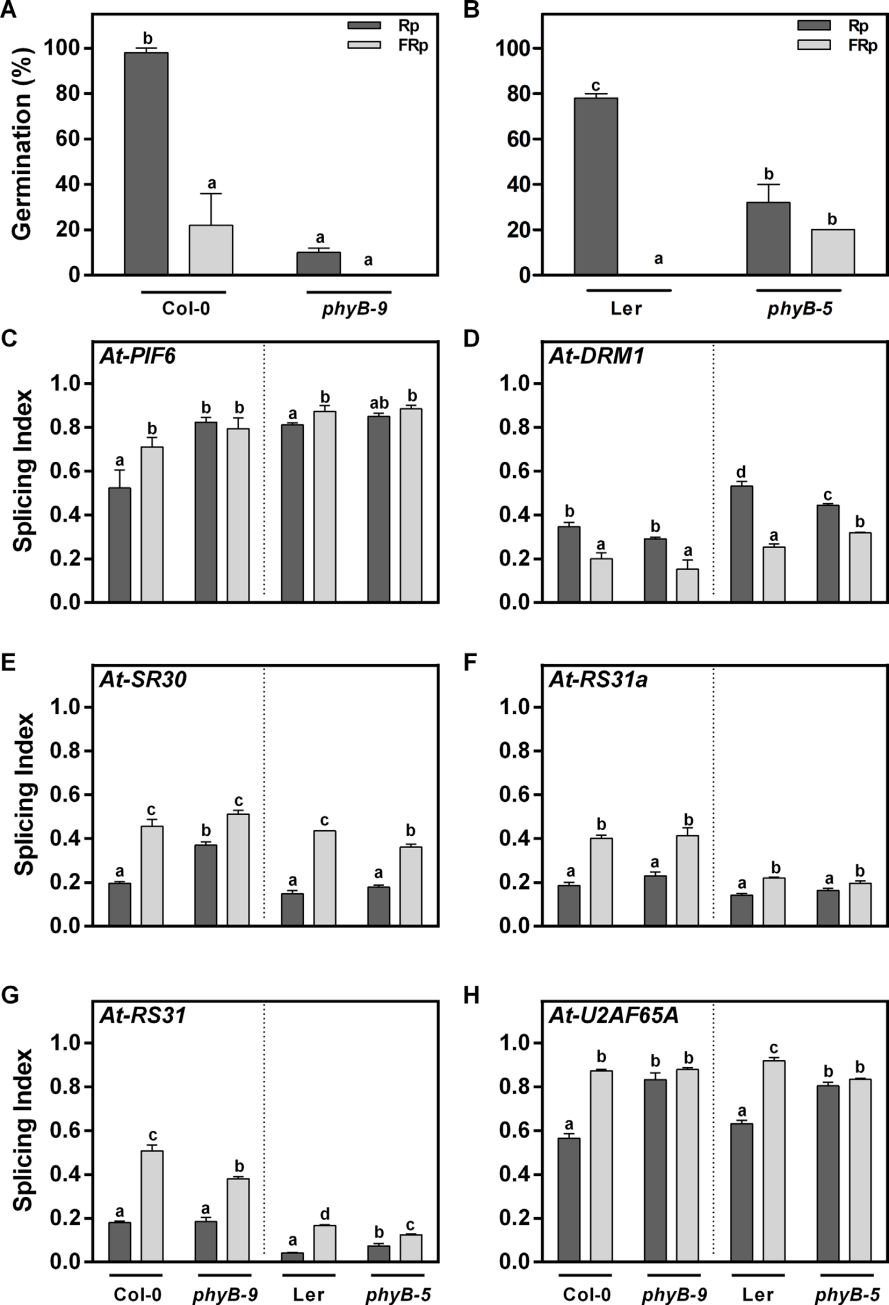
phyB differentially regulates the AS pattern of *At-U2AF65A* and *At-PIF6*. **(A–B)** Germination percentages of Col-0, *phyB-9*, L*er*, and *phyB-5* irradiated with an Rp or an FRp. **(C–H)** AS alterations quantified as splicing index (SI) in response to the Rp or FRp are shown. Seed samples were harvested 12 h after the Rp or FRp. Splice variants of the indicated genes were analyzed by RT–PCR, and each PCR product was quantified by densitometry using ImageJ. Each bar represents mean ± SE (*n* = 3). Means with the same letters are not significantly different (*p* > 0.05 by ANOVA followed by Fisher post-test). ANOVA, analysis of variance; AS, alternative splicing; Col-0, Columbia-0; FRp, far-red pulse; Rp, red pulse; RT–PCR, reverse transcriptase–polymerase chain reaction.

R-light-induced germination can be mediated by different phytochromes (Botto et al., 1996; Hennig et al., 2002; Arana et al., 2014), and both phyA and phyB are the main photoreceptors. The phyB contribution to AS regulation is still unknown. Thus, we analyzed germination of Col-0 and L*er* WT seeds and *phyB-9* (Col-0 background) and *phyB-5* (L*er* background) mutant seeds under our experimental conditions. The FRp does not promote seed germination in any of the analyzed genotypes (**Figures 3A, B**). On the contrary, the Rp induces 98% of germination in Col-0 seeds (**Figure 3A**) and 78% in L*er* seeds (**Figure 3B**), while *phyB-9* and *phyB-5* mutant seeds germinate at 10% and 32%, respectively, suggesting that phyB is the main phytochrome controlling seed germination under these conditions (**Figures 3A, B**).

We then asked whether the light-induced AS changes could be subjected to phyB regulation. The Rp reduces the SI of *At-PIF6*, a phytochrome interacting factor, and increases the SI of *At-DRM1* (*DORMANCY-ASSOCIATED PROTEIN 1*, *AT1G28330*), a dormancy-related gene in Col-0 and L*er* seeds (**Figures 3C, D**). The phyB is responsible for the AS changes of *At-PIF6* since light differences associated with the SI are lost in both *phyB-5 and phyB-9* mutant seeds (**Figure 3C**). In opposition, the AS pattern of *At-DRM1* is not regulated by the phyB (**Figure 3D**). With respect to the splicing related factors, the SI of *At-SR30*, *At-RS31a*, and *At-RS31* are significantly reduced in response to the Rp in both Col-0 and L*er* seeds. Even though *phyB* mutants show some changes in the SI of these AS events, similar differences between Rp and FRp are still observed in both *phyB-9* and *phyB-5* mutant seeds, suggesting the AS control of these splicing factors by light does not involve phyB regulation (**Figures 3E–G**). On the other hand, even though the Rp significantly changes the SI of *At-U2AF65A* in Col-0 and L*er* seeds (∼30%), this difference is completely abolished in the *phyB* mutant seeds in both genetic backgrounds (**Figure 3H**). We conclude that phyB regulates the AS pattern of *At-U2AF65A* and *At-PIF6* in *Arabidopsis* seeds germinating with an Rp.

### Red Light Perception and Alternative Splicing Are Directly Linked

**Figure 4:**
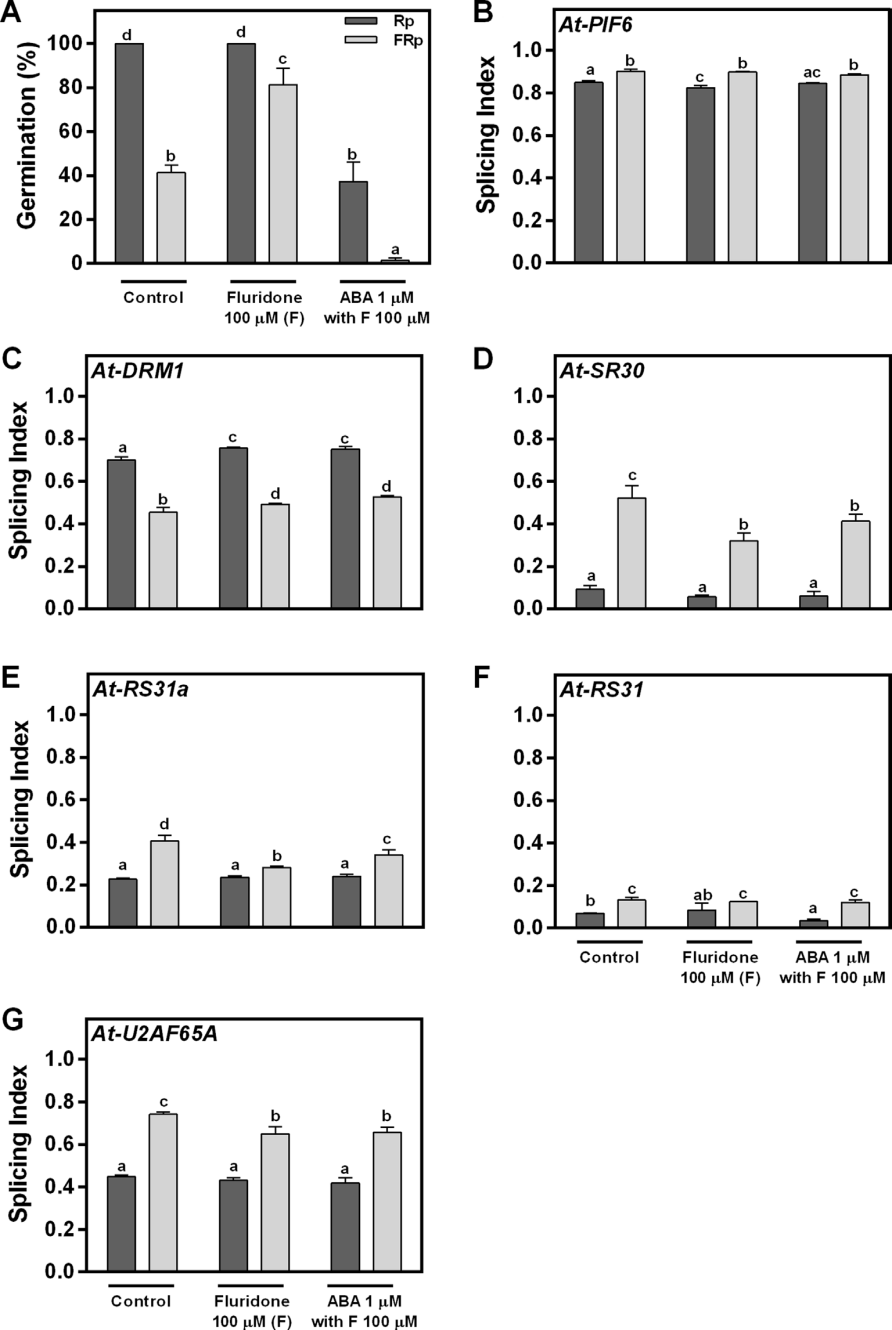
Red light perception and alternative splicing are directly linked. **(A)** Germination percentages of Col-0 seeds imbibed in water (control), fluridone, or ABA supplemented with fluridone and irradiated with an Rp or an FRp. **(B–G)** AS alterations for each hormone condition quantified as splicing index (SI) in response to the Rp or FRp are shown. Seed samples were harvested 12 h after the Rp or FRp. Splice variants of the indicated genes were analyzed by RT–PCR, and each PCR product was quantified by densitometry using ImageJ. Each bar represents mean ± SE (*n* = 3). Means with same letters are not significantly different (*p* > 0.05 by ANOVA followed by Fisher post-test). ABA, abscisic acid; ANOVA, analysis of variance; AS, alternative splicing; Col-0, Columbia-0; FRp, far-red pulse; Rp, red pulse; RT–PCR, reverse transcriptase–polymerase chain reaction.

We have clearly shown that red light promotes seed germination and affects AS (**Figures 1**–**3**). One remaining question is whether these AS changes are directly triggered by the red-light signal or if they are a consequence of the germination process *per se*. We hypothesized that if light is directly controlling the AS response, changes in splicing patterns should not be affected by different levels of germination under the same light condition (Rp or FRp). Since ABA is a known hormone regulating seed germination, we sowed WT Col-0 seeds in water (control); fluridone (F), an inhibitor of ABA synthesis; and fluridone supplemented with ABA (F + ABA). The Rp induces 100% germination in water and F but only 40% in F + ABA. The FRp reduces the germination to ∼40%, ∼80%, and 0%, respectively (**Figure 4A**). As expected, the F and F + ABA treatments modulate the germination response induced by red light. Hence, we used these seeds to analyze the AS patterns of *At-PIF6*, *At-DRM1*, *At-SR30*, *At-RS31a*, *At-RS31*, and *At-U2AF65A*. We found that the SI of the six genes changes significantly between seeds exposed to Rp and FRp in water, and these changes in the SI are still present in F and F + ABA-treated seeds (**Figures 4B–G**). These results clearly show that changes in AS patterns induced by light are independent of the level of germination. This conclusion is also supported by our previous results showing that the *phyB* mutant, which presents reduced germination under Rp, displayed similar AS changes than the WT seeds for the vast majority of the analyzed events (**Figure 3**). Taken altogether, our data suggest that the Rp directly controls AS processes and that these effects are independent of the level of germination induced by light.

## Discussion

Seed dormancy and germination are processes of extreme relevance for a successful seedling establishment in the field. Light is one of the most important and best characterized environmental factors involved in the relief of seed dormancy to promote seed germination (Benech-Arnold et al., 2000). Plants possess a wide variety of photoreceptors capable of sensing this environmental cue (van Gelderen et al., 2018). The *Arabidopsis* genome encodes for five different phytochromes, and phyB is the one with the most prominent role in controlling the R/FR reversible response inducing seed germination (Shinomura et al., 1994; Botto et al., 1995). Moreover, light shapes plants’ transcriptomes by affecting every possible level of gene expression regulation (Petrillo et al., 2015; Merchante et al., 2017; Kaiserli et al., 2018; Godoy Herz et al., 2019). AS, a powerful mechanism that allows rapid changes in transcriptome and proteome complexity during development and in response to changes in the environment (Reddy et al., 2013), is also dramatically affected by this environmental cue at different developmental stages. Nowadays, it is becoming evident that AS substantially increases transcriptome complexity and plays an important role in modulating gene expression in response to internal and external cues (Lightfoot et al., 2008; Matsumura et al., 2009; Sanchez et al., 2010; Martin-Trillo et al., 2011; James et al., 2012; Jones et al., 2012; Rosloski et al., 2013). While the information concerning the molecular basis of light-induced germination is abundant at the level of gene expression/transcription (Penfield et al., 2005; Oh et al., 2006; Oh et al., 2007; Oh et al., 2009; Gabriele et al., 2010; Park et al., 2012; Ibarra et al., 2013), that concerning global studies describing light effects on AS has only recently started to appear. Here, we provide evidence showing that (i) Rp triggers AS changes in some splicing factors, light-signaling components, and dormancy/germination regulators; (ii) Rp exerts these effects on AS through the action of phyB in some few events; and (iii) AS events are regulated by R/FR light, but this regulation is not directly associated with the intensity of germination response.

We identified 226 genes with AS events regulated by light (**Figure 1C** and **Supplementary Table 1**) and that the Rp reduces the SI of *At-SR30*, *At-RS31a*, *At-RS31*, and *At-U2AF65A* (**Figure 2**). Since *At-SR30*, *At-RS31*, and *At-RS31a* are members of the RS subfamily (like *At-RS41*), these results may have implications for all the SR genes in *Arabidopsis*. Previous studies have evaluated light effects on AS at a global level in *Physcomitrella patens* and in etiolated *A. thaliana* seedlings using RNA-seq (Shikata et al., 2014; Wu et al., 2014). These studies found several hundreds of light-regulated AS events, many of which were associated with genes encoding splicing factors and light-signaling components. Interestingly, in both reports, the effects of brief light treatments on AS were modulated to a great extent, although not exclusively, by the phytochromes (Shikata et al., 2014; Wu et al., 2014). Furthermore, it was previously shown that *RRC1* (*REGULATOR OF CHROMOSOME CONDENSATION 1*), an SR-like protein, is required for normal seedling development under R light (Shikata et al., 2014) and, more recently, that RRC1 interacts with SFPS (SPLICING FACTOR FOR PHYTOCHROME SIGNALING) and phyB to coordinately regulate the splicing of genes involved in light signaling and circadian clock pathways to promote photomorphogenesis in *A. thaliana* (Xin et al., 2019). The fact that we found common genes whose AS is regulated in different developmental processes (Shikata et al., 2014; Hartmann et al., 2016; Srinivasan et al., 2016; Narsai et al., 2017; this study) strongly points towards the existence and relevance of an AS regulatory network that is active throughout the whole life cycle of a plant.

The R/FR reversible response of seed germination is mainly mediated by phyB (Shinomura et al., 1994; Botto et al., 1995). Interestingly, we found that changes on some AS events (*At-PIF6* and *At-U2AF65A* out of six genes analyzed) are mediated by this photoreceptor (**Figures 3C, H**). However, the observation that the SI values of *At-SR30*, *At-RS31a*, *At-RS31*, and *At-DRM1* were not altered by the absence of phyB suggests that other stable phytochromes, mediating the R/FR response, might be responsible for the regulation of the AS pattern of these genes. Interestingly, *At-U2AF65A* and *At-RS31* AS is regulated by a common light pathway in seedlings, involving chloroplast’s retrograde signals (Petrillo et al., 2014). Hence, these results suggest seeds can have completely different gene regulatory networks. Further research is needed to know whether other photoreceptors, or other signaling pathways (i.e., retrograde signals from organelles), could be involved in the control of AS patterns in light-germinating seeds. Moreover, since red light promotes seed germination (**Figure 1B**), we could argue that AS changes are a consequence of the germination process. However, we provide compelling evidence showing that Rp and FRp can directly affect AS regulation since the analyzed AS changes are not correlated with the intensity of germination response (**Figure 4**). As previously shown, retrograde signals arising from the organelles are important for triggering AS changes during the transition from darkness to light in *Arabidopsis* seedlings (Petrillo et al., 2014; Riegler et al., 2018). Taking this into account, it is possible to think that retrograde signals arising from different organelles could be associated with signals from photoreceptors to fine-tune the AS process in some genes during light induction of seed germination.

Finally, we conclude that AS is a source of gene expression diversity that potentially leads to different proteins involved in the promotion of seed germination by light, and a proper regulation of this process might be of key relevance for the adjustment of seed germination in the correct place and time. The next step would be to determine which signaling pathway(s) is (are) controlling the splicing of these events and identify the targets of the splicing factors regulated by light. Moreover, the question whether these changes in the AS of different splicing regulators are physiologically relevant is still open. Unraveling these AS regulatory networks will help us understand the mechanisms through which these genes may be responsible in the promotion of germination in crop seeds.

## Data Availability

All datasets for this study are included in the manuscript and the Supplementary files. The RNA-seq data is available at the Gene Expression Omnibus (GEO) with this accession GSE134019

## Author Contributions

EP and JB conceived the project. RT, LS, and EP performed the experiments. CH and MY processed the transcriptome data. MS-S performed the GO analyses. RT, EP, and JB analyzed and interpreted the data and wrote the manuscript. All authors read and approved the final version of the manuscript.

## Funding

The research was supported by Agencia Nacional de Promoción Científica y Tecnológica of Argentina and the University of Buenos Aires (UBA) grants to JB (UBACYT 20020170100265BA and PICT2014-1074) and to EP (PICT2016-4366). RT, CH, and MS-S are fellows from Consejo Nacional de Investigaciones Científicas y Técnicas (CONICET). JB and EP are career investigators from CONICET. LS’s work was supported by the University of Buenos Aires (UBA undergraduate fellowship) and is currently a PhD fellow from CONICET.

## Conflict of Interest Statement

The authors declare that the research was conducted in the absence of any commercial or financial relationships that could be construed as a potential conflict of interest.

## Supplementary Material

Supplementary Figure 1 Calibration curves for the hormone experiments.

Supplementary Table 1 Differential gene expression and alternative splicing analyses.

Supplementary Table 2 Primers used in the study.

Supplementary File 1 Gene models and gel images.
