# Supplementary figures and images for: Alternative Splicing Regulation During Light-Induced Germination of Arabidopsis thaliana Seeds
Source: Front Plant Sci. 2019 Sep 10;10:1076. doi: 10.3389/fpls.2019.01076 (PMC6746916; doi:10.3389/fpls.2019.01076)

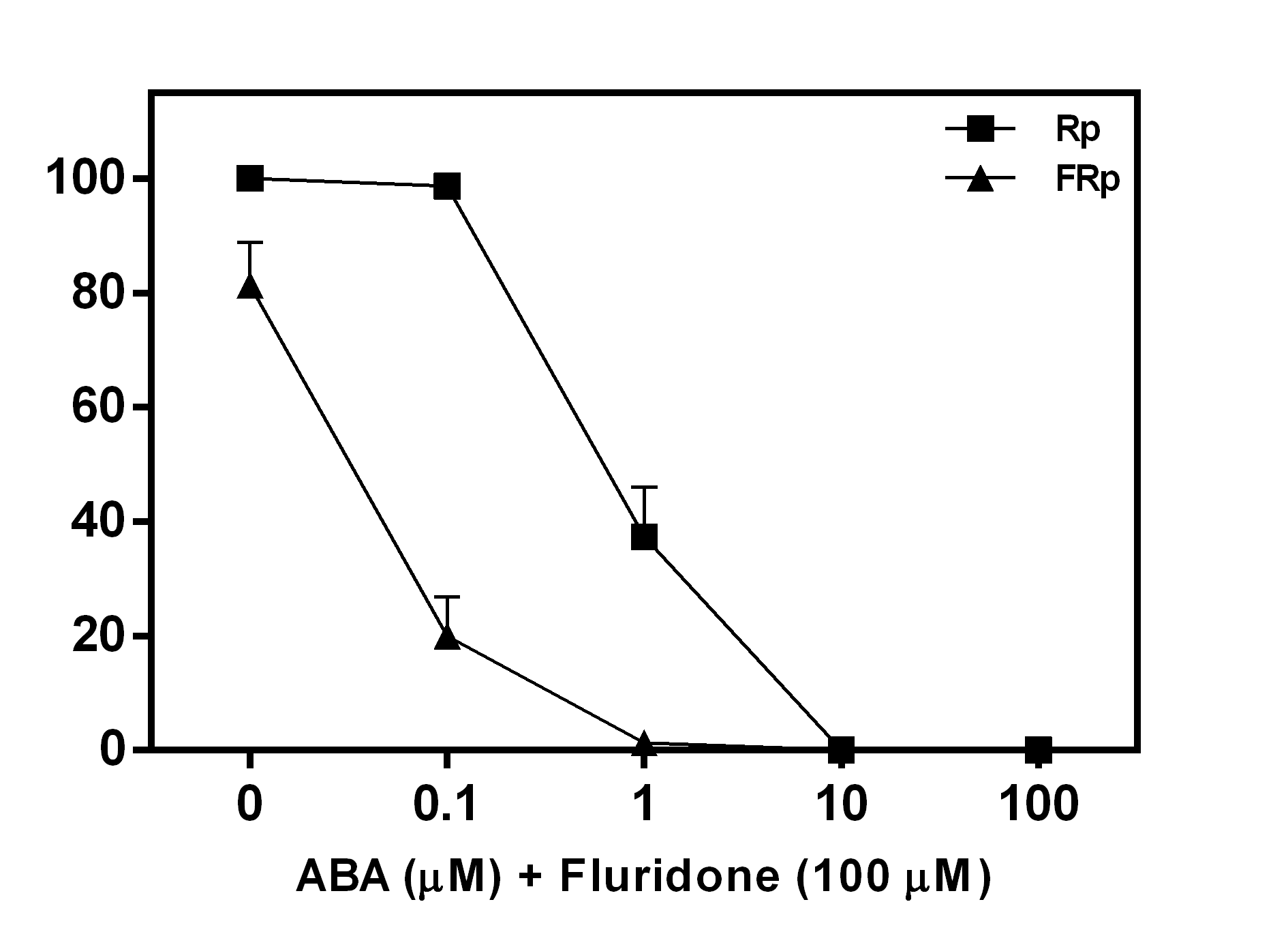

Supplement: Supplementary Figure 1 — Calibration curves for the hormone experiments. [file Image_1.tif]
