# Supplementary material for: Alternative Splicing Regulation During Light-Induced Germination of Arabidopsis thaliana Seeds
Source: Front Plant Sci. 2019 Sep 10;10:1076. doi: 10.3389/fpls.2019.01076 (PMC6746916; doi:10.3389/fpls.2019.01076)
Supplement: Supplementary File 1 — Gene models and gel images. [file DataSheet_1.pdf]

Gene Models, Locations of the primers used for the splicing analyses and representative Gel Images

At-PIF6

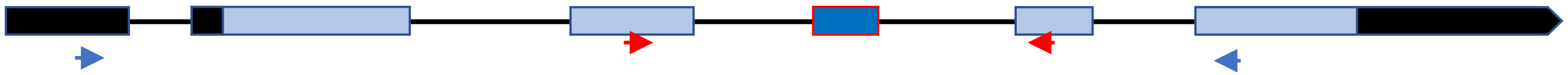

Gene Model

Red arrows represent the primers
Blue arrows show the primers used for the long PCR
Boxes represent exons
Lines represent introns

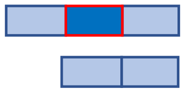

Isoforms
(short RT-PCR products)

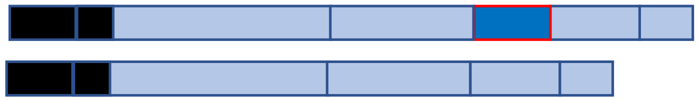

(long RT-PCR products)

Representative gel images corresponding to Figure 3

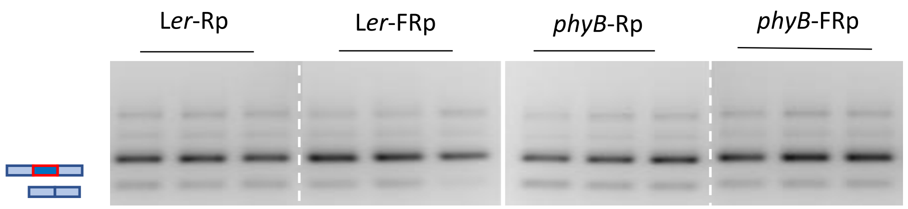

Representative gel image corresponding to Figure 4

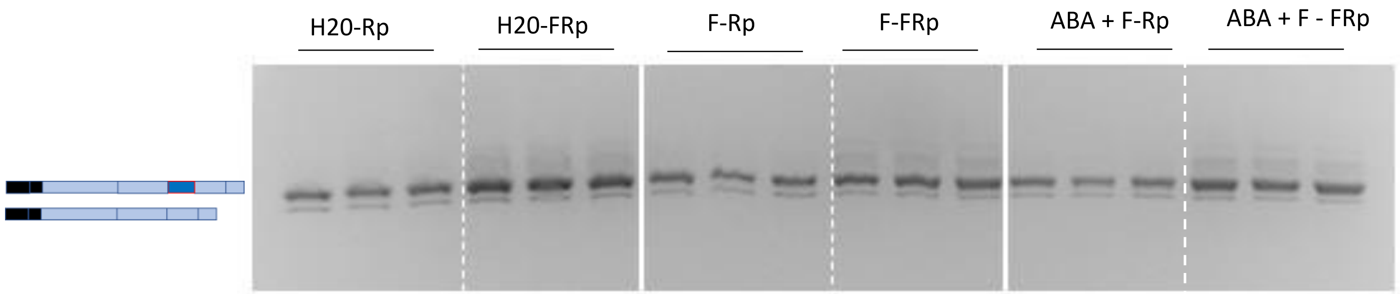

Each treatment consists of 3 biological replicates
Rp = Red Pulse
FRp = Far Red Pulse
F = fluridone

At-DRM1

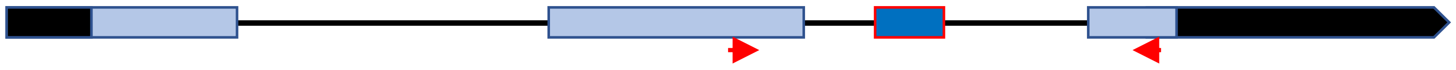

Gene Model

Red arrows represent the primers
Boxes represent exons
Lines represent introns

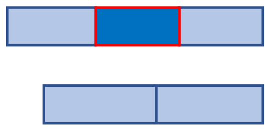

Isoforms
(RT-PCR products)

Representative gel images corresponding to Figure 3

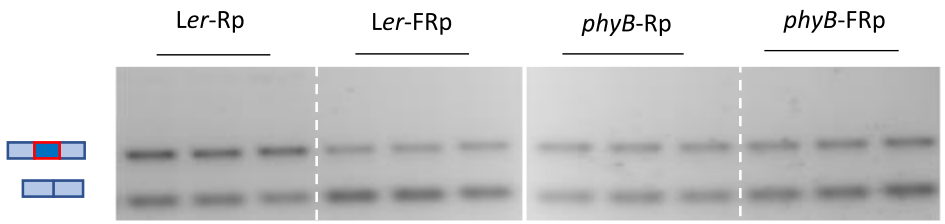

Representative gel image corresponding to Figure 4

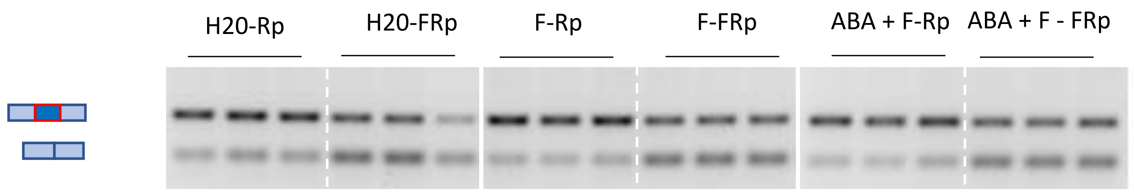

Each treatment consists of 3 biological replicates
Rp = Red Pulse
FRp = Far Red Pulse
F = fluridone

At-SR30

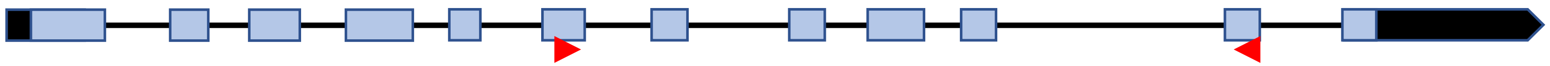

Gene Model

Red arrows represent the primers
Boxes represent exons
Lines represent introns

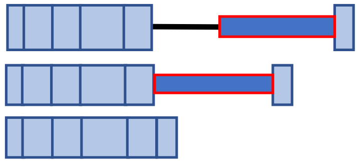

Isoforms
(RT-PCR products)

Representative gel images corresponding to Figure 3

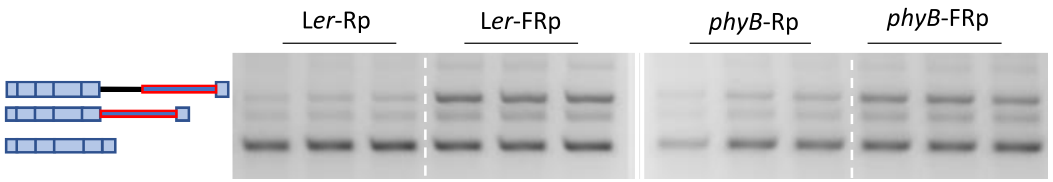

Representative gel image corresponding to Figure 4

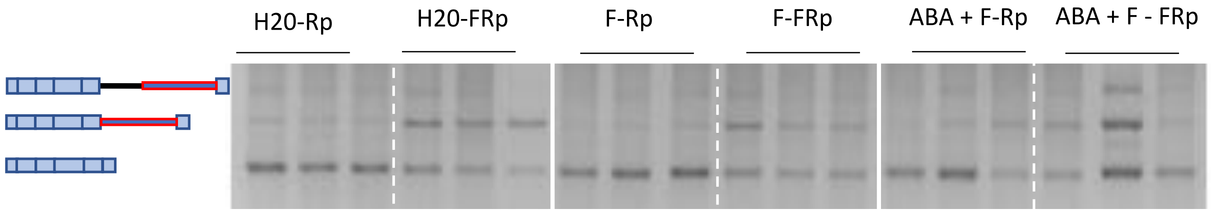

Each treatment consists of 3 biological replicates
Rp = Red Pulse
FRp = Far Red Pulse
F = fluridone

At-RS31a

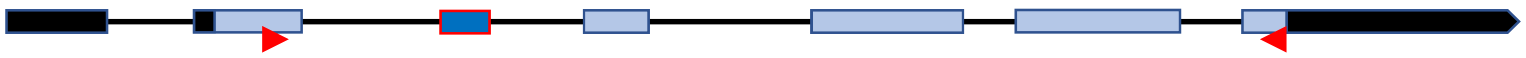

Gene Model

Red arrows represent the primers
Boxes represent exons
Lines represent introns

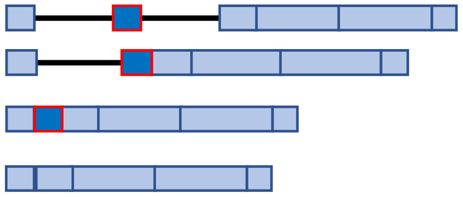

Isoforms
(RT-PCR products)

Representative gel images corresponding to Figure 3

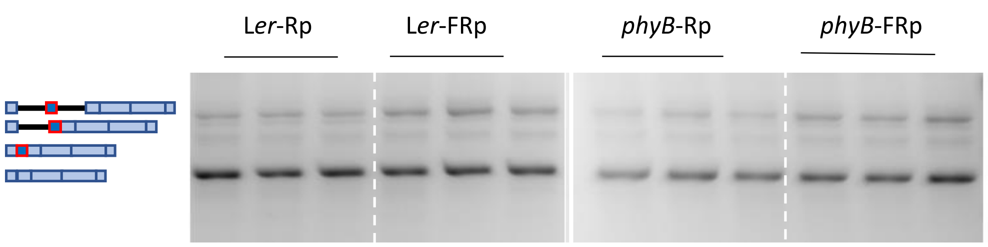

Representative gel image corresponding to Figure 4

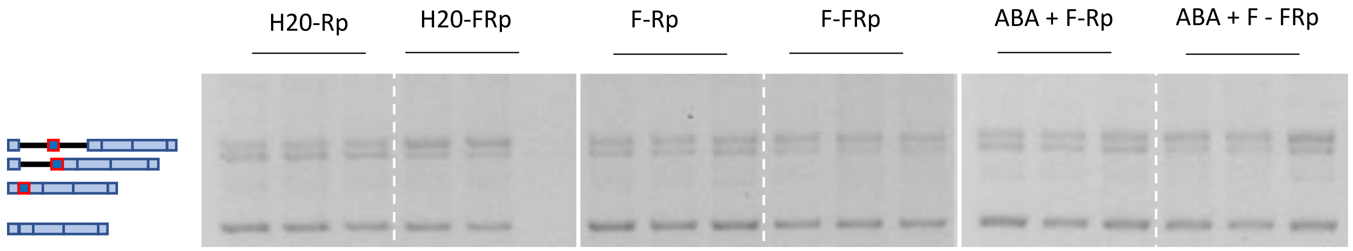

Each treatment consists of 3 biological replicates
Rp = Red Pulse
FRp = Far Red Pulse
F = fluridone

At-RS31

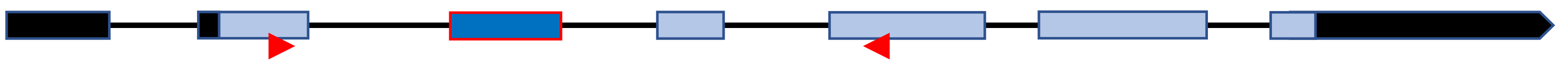

Gene Model

Red arrows represent the primers
Boxes represent exons
Lines represent introns

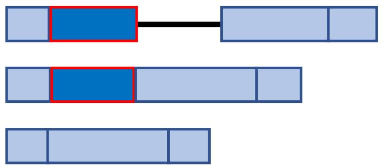

Isoforms
(RT-PCR products)

Representative gel images corresponding to Figure 3

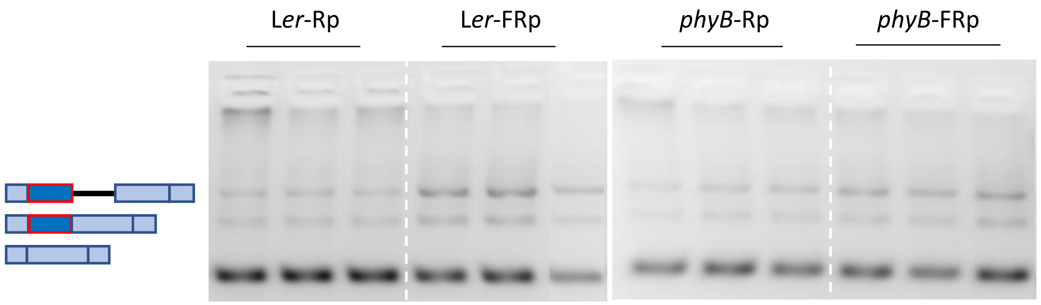

Each treatment consists of 3 biological replicates
Rp = Red Pulse
FRp = Far Red Pulse
F = fluridone

Representative gel image corresponding to Figure 4

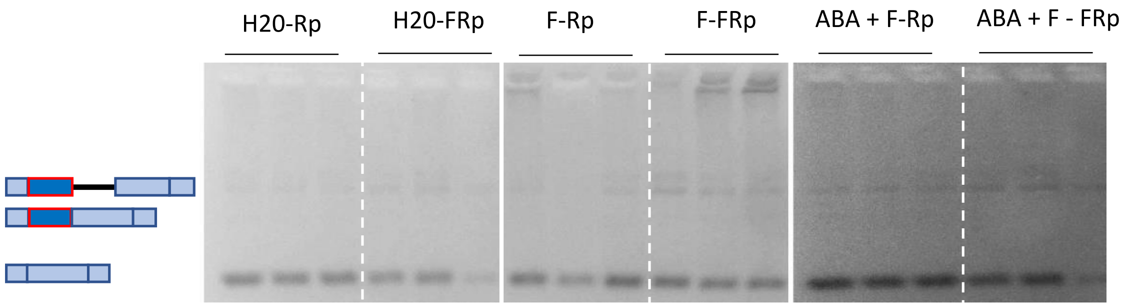

At-U2AF65A

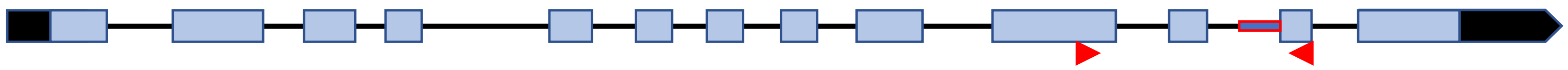

Gene Model

Red arrows represent the primers
Boxes represent exons
Lines represent introns

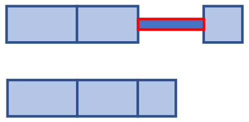

Isoforms
(RT-PCR products)

Representative gel images corresponding to Figure 3

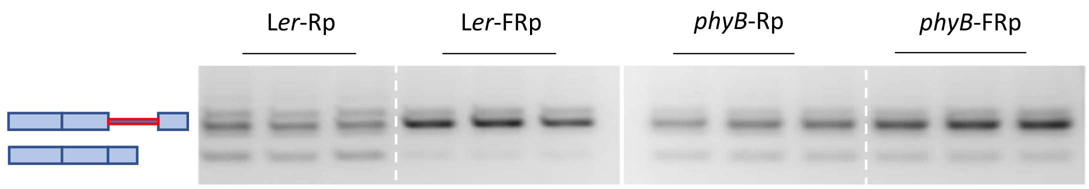

Representative gel image corresponding to Figure 4

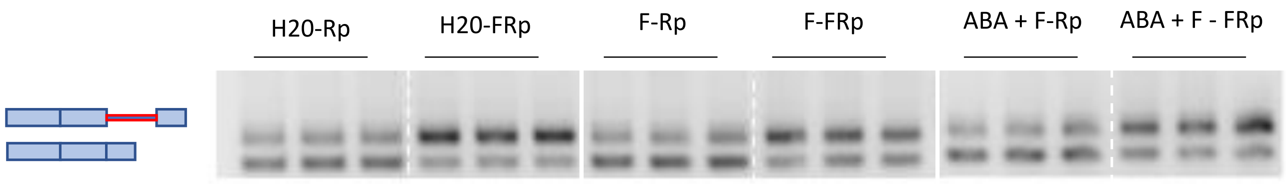

Each treatment consists of 3 biological replicates
Rp = Red Pulse
FRp = Far Red Pulse
F = fluridone
